# Pre‐treatment with *Trichoderma viride*: Towards a better understanding of its consequences for anaerobic digestion

**DOI:** 10.1111/1758-2229.13281

**Published:** 2024-06-28

**Authors:** Rudolf Markt, Eva Maria Prem, Nina Lackner, Mira Mutschlechner, Paul Illmer, Andreas Otto Wagner

**Affiliations:** ^1^ Department of Microbiology Universität Innsbruck Innsbruck Austria

## Abstract

Understanding and optimising biological pre‐treatment strategies for enhanced bio‐methane production is a central aspect in second‐generation biofuel research. In this regard, the application of fungi for pre‐treatment seems highly promising; however, understanding the mode of action is crucial. Here, we show how aerobic pre‐treatment of crystalline cellulose with the cellulolytic *Trichoderma viride* affects substrate degradability during mesophilic, anaerobic digestion. It could be demonstrated that fungal pre‐treatment resulted in a slightly reduced substrate mass. Nevertheless, no significant impact on the overall methane yield was found during batch fermentation. Short chain organic acids accumulation, thus, overall degradation dynamics including methane production kinetics were affected by the pre‐treatment as shown by Gompertz modelling. Finally, 16S rRNA amplicon sequencing followed by ANCOM‐BC resulted in up to 53 operative taxonomic units including fermentative, syntrophic and methanogenic taxa, whereby their relative abundances were significantly affected by fungal pre‐treatment depending on the duration of the pre‐treatment. The results demonstrated the impact of soft rot fungal pre‐treatment of cellulose on subsequent anaerobic cellulose hydrolysis as well as on methanogenic activity. To the best of our knowledge, this is the first study to investigate the direct causal effects of pre‐treatment with *T. viride* on basic but crucial anaerobic digestion parameters in a highly standardised approach.

## Graphical abstract

Crystalline cellulose was biologically pre‐treated with a pure culture of the fungus *Trichoderma viride* under aerobic conditions. In a subsequent anaerobic digestion step methane production kinetics, cellulose degradation and methanogenic activity but not total methane yield were affected.

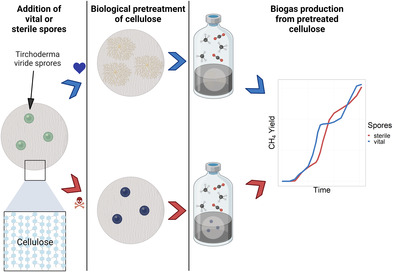

## INTRODUCTION

Anaerobic digestion (AD) of organic feedstock for the conversion into biogas is a microbes driven process to produce renewable bio‐methane and substitute fossil energy carriers (Ellabban et al., 2014). The political promotion of AD resulted in an extensive cultivation of energy crops to meet the increasing demand for organic feedstock, especially in Europe's biogas leader Germany (Brémond et al., 2018). The use of energy crops for biofuel production (first‐generation fuels) is discussed controversially as it causes biodiversity issues and competes with food production. This encourages scientists to rethink feedstock sources and focus on non‐food plant biomass (second‐generation biofuels) which on top are abundant and cheap (Naik et al., 2010).

However, these socioeconomically and ecologically preferable substrates, such as lignocellulose (Karrabi et al., 2023) or long chain fatty acids (Liu et al., 2023), are often difficult to degrade for microorganisms. Chemical, physical and biological pre‐treatment technologies can enhance biogas production from these feedstocks and are thus inevitable (Shrestha et al., 2017; Wagner et al., 2018; Zheng et al., 2014). While physical and chemical pre‐treatment methods demand high chemical and/or energy input (Karrabi et al., 2023), biological pre‐treatment methods employ biogenic catalysts—microorganisms or enzymes—to enhance substrate degradability at considerably milder operation conditions (Sindhu et al., 2016; Wagner et al., 2018). However, growth and feedstock consumption rates of microorganisms involved in pre‐treatment may limit the efficiency of biological pre‐treatments (Wagner et al., 2018); besides, environmental requirements for an effective application can be challenging to meet and maintain (Karrabi et al., 2023). Nevertheless, recent publications showed the enormous potential of such methods, especially when applying lignolytic white rot fungi (Kainthola et al., 2019; Liu et al., 2017; Zhao et al., 2014): *Ceriporiopsis* sp., *Phanerochaete* sp., *Fusarium* sp., *Trametes* sp., *Pleurotus* sp.*—*among others—could decrease lignin contents by 17%–70% and increase methane production by 20%–400% (Ge et al., 2015; Liu et al., 2014; Tišma et al., 2018; Zhao et al., 2014).

Apart from white rot fungi, the applicability of cellulolytic soft rot fungi as biological pre‐treatment agents was also investigated: The cellulolytic enzymes of *Trichoderma viride* reduced the crystallinity of cellulose and convert its structure to a more amorphous state (Popescu et al., 2010). Indeed, under overload conditions the pre‐treatment of bio‐waste with *T. viride* led to an increased biomethane production (Mutschlechner et al., 2015; Wagner et al., 2013). In another study, Muthangya et al. successfully combined soft rot and white rot fungi to increase methane potential from lignocellulosic waste (Muthangya et al., 2009). However, due to the heterogeneity of the investigated substrates, the mode of action of soft rot fungi as biological catalysts for cellulose pre‐treatment as well as the impact on the downstream anaerobic digestion process is not yet fully elucidated.

Therefore, the objective of this study was to investigate: (i) fungal growth and cellulolytic activity of a pure culture of *T. viride* while pre‐treating highly crystalline cellulose under sterile conditions, (ii) the effect of pre‐treatment of highly crystalline cellulose with *T. viride* on the subsequent biomethane production, formation of intermediate fermentation products as well as on the anaerobic microbial community.

## EXPERIMENTAL PROCEDURES

### Biological pre‐treatment of cellulosic folded filters

Cellulose based folded‐filters (MN 615 ¼, Macherey&Nagel, Germany), thereon called FF, with a dry mass of 1.23 ± 0.03 g per filter, were autoclaved and inoculated with vital or double‐autoclaved, sterile spores of *T. viride*. According to the manufacturer, FF were made up of high‐quality cellulose from cotton linter and were pulped with organic solvents. This type of material mainly comprises crystalline α‐cellulose as described previously (Gümüskaya et al., 2003). *T. viride* spores were produced according to an in‐house protocol on a solid matrix (Illmer et al., 2007). After drying, spores were submerged in mineral medium composed of 30 mM KH_2_PO_4_, 82 mM NH_4_NO_3_, 5.5 mM Mg(NO_3_)_2_ × 6 H_2_O, 1 mM ZnSO_4_ × 7H_2_O and 2 mL l^−1^ trace element stock solution. This stock solution contained 540 μM FeSO_4_ × 7H_2_O, 12 μM CuSO_4_ × 5H_2_O and 90 μM MnSO_4_ × 1H2O. The pH of the mineral medium was adjusted to pH 6 using 1 M NaOH.

FF were put in sterile 50 mL falcons with screw caps and inoculated with 1.5 mg of dried spores resuspended in 3 mL mineral medium. For both treatments (sterile or vital spores), nine replicates were incubated at 25°C under aerobic conditions for 0, 3, 6, 10 or 14 days, respectively (9 filter × 5 different incubation time assays). Each time assay started individually to guarantee a simultaneous end of incubation. This procedure was applied to increase comparability among variants during the subsequent AD experiment (please see below). The filters were frozen and freeze‐dried immediately after incubation unless stated otherwise. For each treatment and pre‐treatment timepoint (nine filters), triplicates were used for (i) mass loss, (ii) the paper filter assays (cellulytic activity, leachable carbon and nitrogen) and (iii) the subsequent AD experiments in batch mode.

### Batch experiments for biogas production

A biomethane potential test was performed under mesophilic conditions to analyse methane production as well as its kinetics of pretreated FF. The experimental setup included filters treated with sterile spores (controls) and filters treated with vital spores for all aerobic incubation periods (0, 3, 6, 10 or 14 days). To obtain background methane potential from the inoculum, pure inoculum was tested in triplicate as a negative control as well. Therefore, 33 reactors were set up for this approach.

In detail, 130 mL of anaerobic sludge, obtained from the mesophilic wastewater treatment plant in Zirl, Austria (Aichinger et al., 2015), were sieved to 5 mm and filled into 250 mL Schott® flasks. Each batch reactor was amended with one pretreated FF (except for the negative controls). To maintain anaerobic conditions, reactors were immediately flushed with nitrogen gas for 60 s, closed with butyl rubber septa (Ochs, Germany) and incubated at 37°C for 31 days. The experimental setup guaranteed no overload conditions due to a small substrate to inoculum ratio (S/I ratio) <0.5 based on volatile suspended solids according to (Verein deutscher Ingenieure, 2006); the S/I ratio was 0.473 for the control reactors with no FF degradation. Methane production was measured on day 0, 1, 3, 5, 6, 7, 8, 10, 12, 13, 14, 15, 16, 18, 20, 23, 26, 29 and 31. On day 0, 3, 7, 12, 15, 21, 26 and 31 liquid sample were taken with cannula and syringe for short chain organic acid analyses as well as ergosterol quantification. Liquid samples were stored at −20°C immediately after sampling.

Methane production was monitored by measuring the overpressure with a GHM Greisinger GDH 200 sensor and methane concentrations via GC‐FID (Wagner et al., 2011), and by calculations based on the ideal gas law (Wagner et al. 2019a).

The concentration of fermentation products including short chain organic acids (SCOA), short chain alcohols, and sugars were measured via HPLC‐UV/VIS or HPLC‐RI on a Shimadzu Prominance HPLC System (Shimadzu, Japan) as described previously (Wagner et al., 2017). Sludge samples were stored at −20°C until measurement.

#### Modified Gompertz equation, and energetic calculation

For analysing methane production kinetics, a modified Gompertz equation according to Equation (1) (Lay et al., 1996) was used as described previously (Díaz et al., 2011; Owamah & Izinyon, 2015; Syaichurrozi et al., 2013).

(1) $$P_{t}=P_{m}e^{\left\{-e^{\left[\;\frac{R_{m}}{P_{m}}\left(\lambda -t\right)+1\right]}\right\}}$$

whereby *P*_*t*_ = the cumulative methane amount (NmL g^−1^ FF) at timepoint *t* [*d*]. *P*_*m*_ = the maximal methane amount (NmL g^−1^ FF). *R*_*m*_ = the maximal methane production rate (NmL g^−1^ FF d^−1^). λ corresponds to the initial lag phase (*d*).

Curve fitting was performed using the iterative solver function of Microsoft Excel (Microsoft, USA). The calculation was conducted forcing a minimal root mean square error (RMSE) over the completely anaerobic incubation period, whereby the variables *P*_*m*_ and *t* were measured and were *R*_*m*_ and *λ* were approximated. The quality of the fitted model was depicted as coefficient of determination (*R* ^2^).

To estimate the theoretical methane potential of cellulose, the Buswell‐Boyle equation was applied (Buswell & Mueller, 1952), resulting in a calculated yield of 415 NmL methane per g pure α‐cellulose (C_6_H_10_O_5_)_n_. Mass loss of folded filters was calculated based on dry mass estimation after freeze drying.

### Paper filter assays

To leach soluble compounds from FF, 24 mL distilled water were added to the filters which were subsequently shaken using an overhead shaker at 30 rpm for 30 min. The resulting suspension was centrifuged at 20000×g for 10 min, sterile‐filtered with 0.20 μm RC syringe filters (sterile, Phenomenex, Germany), and diluted 26‐fold in sterile distilled water. Elemental analysis regarding soluble total carbon (sTC), soluble non‐purgeable organic carbon (sNPOC) and soluble total nitrogen (sTN) from leached FF were quantified using a Shimadzu TOC analyser (Shimadzu, Japan) according to the protocol of the manufacturer and previous analyses (Wagner, Prem, et al., 2019).

Filter paper assays for cellulolytic activity were prepared according to previous investigations (Chu et al., 2012) and modified as follows: freeze dried folded filters were incubated for 0 and 24 h in 50 mM citrate buffer (pH 4.8) at 50°C, whereby 50 mL of buffer were added to 1.0 g of pretreated filter. Increase in cellobiose and glucose concentration was analysed after 0 and 24 h of incubation and used to calculate enzymatic activity according to Equations (2) and (3) for exocellulase activity and beta‐glucosidase activity, respectively.

(2) $${\text{Cell}}_{\mathrm{exo}}=\frac{{\text{Cell}}_{24}-{\text{Cell}}_{0}+\left({\mathrm{Glu}}_{24}-{\mathrm{Glu}}_{0}\right)*2}{8.64}*5$$

(3) $${\mathrm{Glu}}_{ß}=\frac{{\mathrm{Glu}}_{24}-{\mathrm{Glu}}_{0}}{8.64}*5$$

whereby Cell_exo_ = exocellulase activity in (μmol s^−1^ g FF^−1^). *glu*_*ß*_ = ß‐glucosidase activity (μmol s^−1^ g FF^−1^).

*Cell*_*0*_ or *Cell*_*24*_ and *Glu*_*0*_ or *Glu*_*24*_ stand for measured cellobiose and glucose concentration (mM) at the beginning and end of the assays.

Concentrations of cellobiose and glucose, solubilised in citrate buffer, were determined after centrifugation for 10 min at 20000× *g* and sterile‐filtration (0.20 μm PTFE syringe filter, Phenomenex, Germany) by HPLC‐ELSD on a Shimadzu Prominance HPLC System (Shimadzu, Japan). Liquid chromatography was performed on a Rezex™ RPM‐Monosaccharide Pb^2+^ column (Phenomenex, USA) running in isocratic mode with distilled water at 85°C. ELSD was operated with particle free air at 350 kPa at 60°C.

The ergosterol content from freeze dried folded filters was quantified after saponification on a HPLC‐UV/VIS according to Schinner et al. (1996), whereby the extraction solvent was directly injected into the LC‐System (Shimadzu Prominence, Japan).

### Calculation of electron flux

To compare the total electron turnover from cellulose to fermentation products and methane, electron equivalents were calculated based on a complete stoichiometric oxidation of carbon compounds with oxygen to carbon dioxide and water. Hence, 1 mol of consumed oxygen corresponds to 4 electron equivalents (e‐q) (Henze et al., 2008). Thus, a total of 8 e‐q mol^−1^, 14 e‐q mol^−1^, 20 e‐q mol^−1^ and 8 e‐q mol^−1^ for one mol acetic acid, propionic acid, butyric acid and methane, respectively, were used for calculation.

### NGS analysis

A total of 240 samples from the anaerobic phase were chosen for 16S rRNA amplicon sequencing. DNA extraction was done with the Soil Extract II Kit DNA (Macherey‐Nagel) according to manufacturer's protocol. The DNA was eluted in 50 μL elution buffer. DNA quantity and co‐extraction of contaminants was checked via the NanoDrop 2000c™ system. The NGS‐library was created according to previous protocols (Prem et al., 2019) using NEBNext® Ultra™ II Q5® Master Mix (New England Biolabs, Germany) and the primer pair 515f/806r targeting the V4 region (Apprill et al., 2015). The final ready‐to‐load sample pool (15 ng μL^−1^) was sent to Microsynth AG (Switzerland) and sequenced on a MiSeq™ System (Illumina®, USA) according to the company's protocols.

Reads from Illumina sequencing were processed and analysed with the *Dada2* (1.16) pipeline (Callahan et al., 2016), using the SILVA 138.1 database silva_nr99_v138.1_train_set.fa.gz (McLaren & Callahan, 2021; Quast et al., 2013; Yilmaz et al., 2014) for taxa assignment. Forward and reverse reads were trimmed to 240 and 200 bp, respectively, before primer removal. Median read depth was 32,800 reads sample^−1^. Eight samples with a depth below 18,000 reads were removed from the dataset. Further operative taxonomic units (OTUs) which occurred in less than 12 samples were pooled as rare OTUs. For a better understanding of the temporal dynamics of the microbial community composition during the anaerobic digestion of cellulose filters irrespective of the pre‐treatment, a subset was used containing samples without pre‐treatment (sterile spores) and the inoculum itself as starting point. Furthermore, the pre‐treatment effect was analysed by comparing samples treated with sterile or vital spores. Five separate datasets were used, one for every pre‐treatment period. For each dataset, differential OTUs were determined with the ANCOM‐BC algorithm (Lin & Peddada, 2020).

### Further statistical analysis

Statistical analyses were performed using the software package Past 3.25 (Hammer et al., 2001). Non‐parametric Wilcoxon signed‐rank test was applied to test for significant differences between treatment with vital and sterile spores. The pairing variable within test statistics was the aerobic pre‐treatment period excluding the treatments of 0 days of aerobic pre‐treatment. A significance level of *p* < 0.05 and *p* < 0.01 was used to define significantly different and highly significantly different results, respectively. Results are given as mean ± standard deviation from three replicates. Graphical processing was conducted using the software package ‘R’ version 4.2.3 including the libraries *readxl*, *strings*, *reshape2* and *dbplyr* for data handling and *ggplot2* for figure generation (Kolde, 2019; Wickham, 2007, 2022; Wickham et al., 2023; Wickham & Bryan, 2023). The heatmap, showing the differential OTUs examined by the ANCOM‐BC algorithm was done with *pheatmap* after transforming the relative abundance by taking the power of 0.25. *Euclidean* distance measure was used for clustering rows.

## RESULTS AND DISCUSSION

### Fungal activity and pre‐treatment effect on cellulose

**FIGURE 1:**
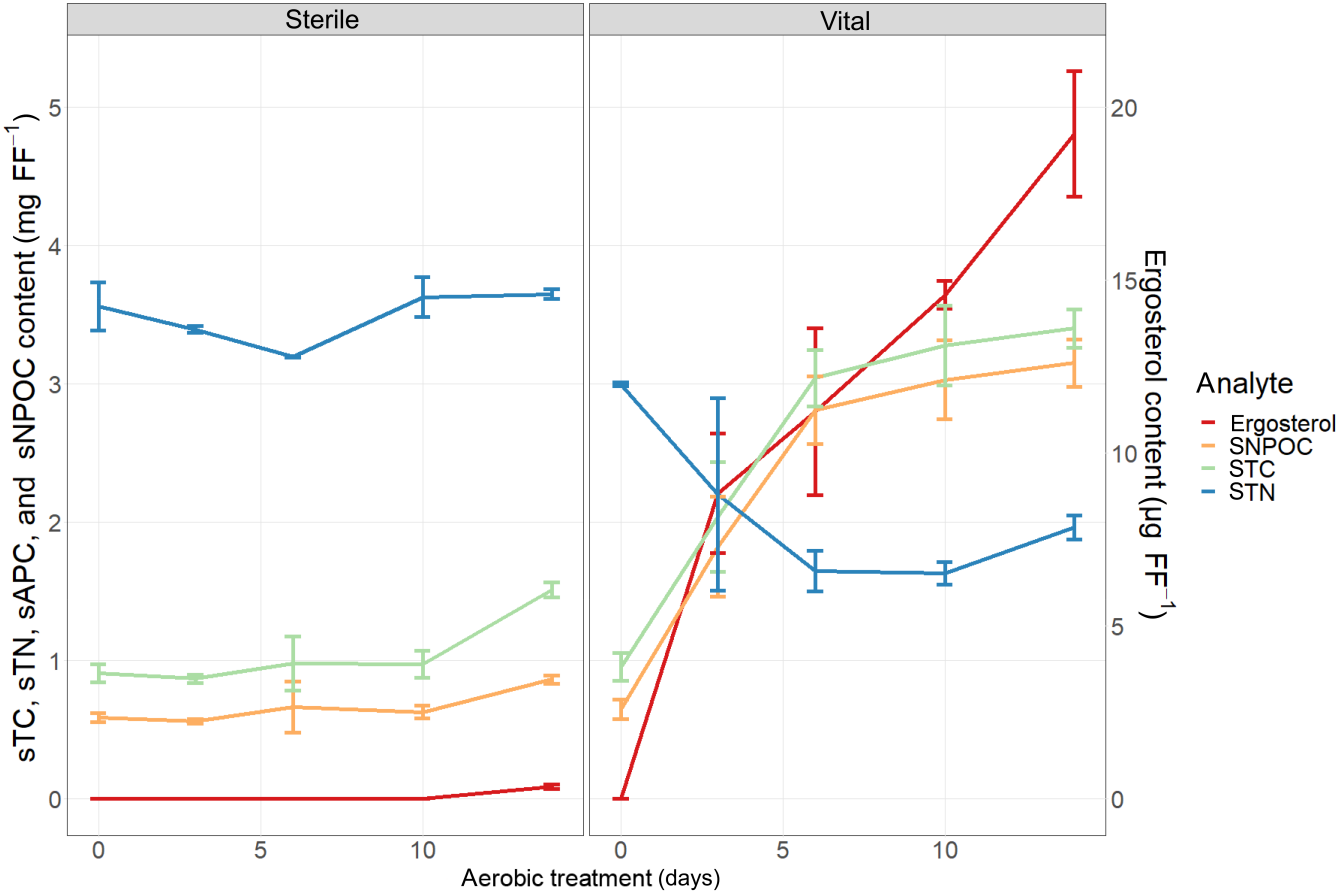
Total carbon (sTC), total nitrogen (sTN), non‐purgeable organic carbon (sNPOC) and ergosterol content during 14 days of aerobic incubation of folded filters (FF) without (sterile) and with (vital) the addition of *Trichoderma viride* spores.

The consumption of dissolved nitrogen as well as the production of ergosterol and the coeval increase of soluble carbon on filters inoculated with vital *T. viride* spores were clear indicators for fungal growth and cellulolytic activity as can be seen in Figure 1. Regarding the ergosterol content as well as the elemental parameters sNPOC, sTC and sTN, differences between treated samples and controls incubated with sterile spores were highly significant. While sTC and sNPOC increased in a parallel manner until day 14, sTN decreased on treated filters until day 6. Contrary, sTC, sNPOC and sTN in the controls remained nearly stable. According to these results, high fungal activity occurred until day 6 followed by a decelerated activity indicated by stagnating sTN consumption and sNPOC and sTC amount. The ergosterol concentration, however, increased in a linear manner from day 3 until the end of the pre‐treatment period on filters treated with vital spores. Thereby, ergosterol is an indicator for fungal biomass production (West et al., 1987) and was used here to prove fungal growth. In contrast, the ergosterol content in the control filters remained low and derived from sterilised spores. High cellulolytic activity of *T. viride* during biological pre‐treatment was also shown previously (Mutschlechner et al., 2015) and was proven in the present study by measuring the enzymatic exocellulose and ß‐glucosidase activity on filters treated with vital spores. According to the filter paper assay, the cellulolytic enzymes showed high rates of substrate conversion, producing 0.30 ± 0.05 μkat g^−1^ FF cellobiose and 0.35 ± 0.10 μkat g^−1^ FF glucose in samples taken after 3 days of aerobic incubation. This level of enzymatic activity was roughly stable until day 14 of the aerobic incubation (data not shown) which presumably led to a decrease in cellulose crystallinity; this was also shown in a previous investigation (Popescu et al., 2010). Relative mass loss of lyophilised filters treated with vital spores compared with control filters reached a maximum of 67 ± 37 mg FF^−1^ after 14 days of aerobic incubation. This weight reduction of filters can be attributed to the catabolic metabolism of *T. viride* and was deliberately not compensated during downstream analysis.

Concluding, fungal growth and cellulolytic activity could be proven during the aerobic pre‐treatment of cellulose with a pure culture of *T. viride*. Contrary, control filters were not affected by the aerobic incubation with sterile spores.

### Influence of pre‐treatment on downstream anaerobic degradation

#### Methane yield and methane production kinetics

After aerobic incubation, cellulose filters (treated with vital or sterile spores) were anaerobically digested under mesophilic condition over a period spanning 31 days. Thereby, mean cumulative methane production reached a final level of 494 ± 16 NmL flask^−1^ over all treatments. Hence, methane yields were not significantly different between filters treated with vital or sterile spores. The small mass loss of filters due to fungal pre‐treatment did not affect methane yield. To estimate the specific bio‐methane potential of the folded filters, the methane yield from the control reactors (without substrate) (114 ± 8 NmL flask^−1^) was subtracted from the total methane yield and scaled to 1.0 g filter, resulting in a specific biomethane potential of approximately 317 NmL g^−1^ FF. Calculated according to Buswell‐Boyle equation (Buswell & Mueller, 1952), 1.0 g cellulose (C_6_H_10_O_5_)_n_ corresponds to a theoretical specific methane yield of 415 NmL g^−1^ FF during complete catabolic conversion. Therefore, the final specific methane amount of 317 NmL g^−1^ FF is equivalent to a relative catabolic conversion of more than 75%.

**FIGURE 2:**
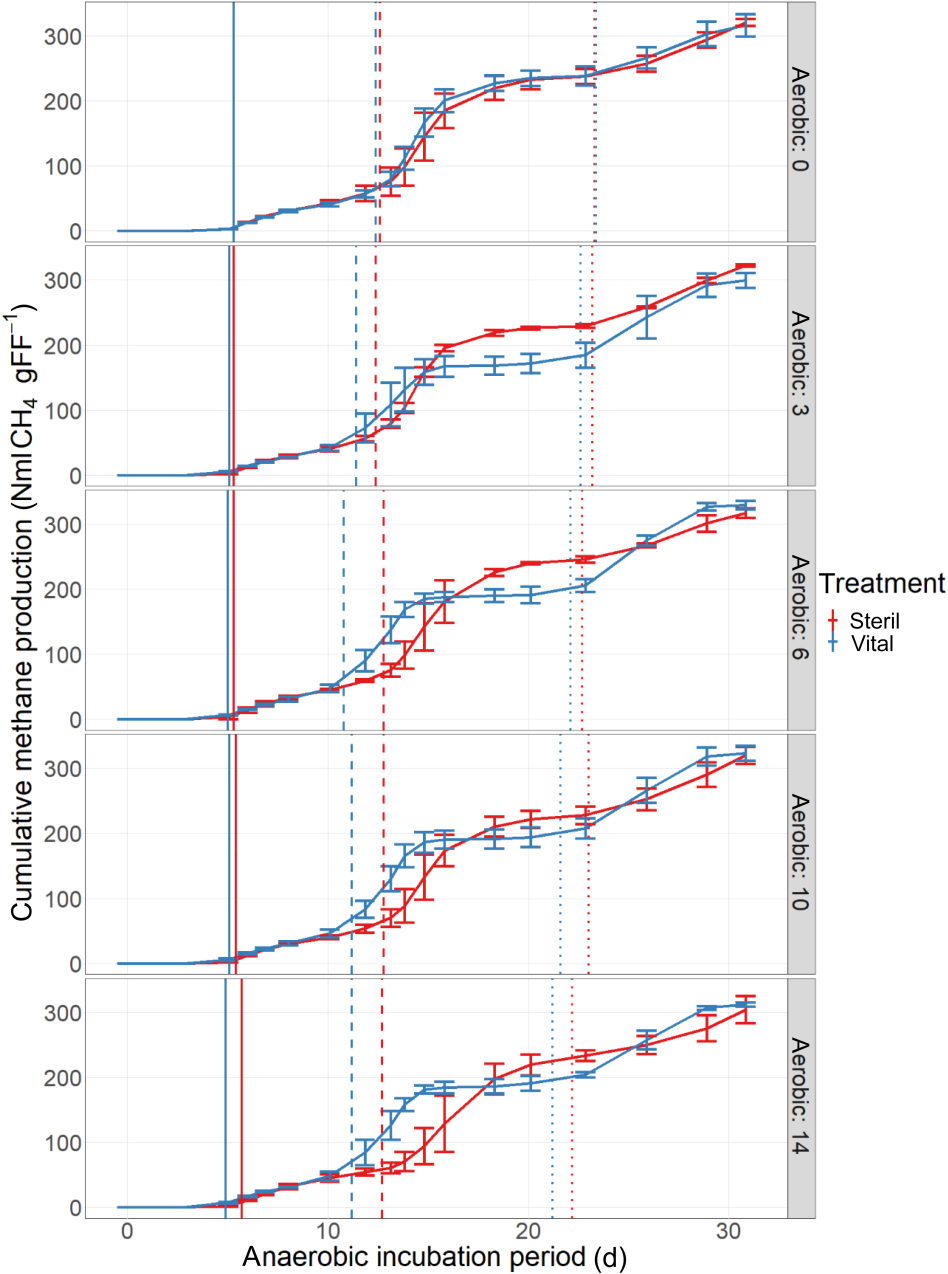
Cumulative methane production of not (sterile) and pre‐treated (vital) substrate. Prior to anaerobic digestion folded filters of vital treatment were aerobically incubated with *Trichoderma viride* for 0, 3, 6, 10 and 14 days. Solid, dashed and dotted lines inidcate the start of the first, second and third biogas production phase, respectively.

In contrast to the overall methane yield, methane production kinetics were strongly affected by the upstream aerobic fungal pre‐treatment. As depicted in Figure 2, the cumulative methane production curves differed between controls and filters treated with vital spores. To analyse this effect in more detail, a curve fitting of the course of the cumulative methane production was performed using a modified Gompertz equation. The methane production kinetics underwent a rather polyphasic than monophasic progression as observed previously for batch biogas production (Buitrón et al., 2019; Turick et al., 1991). These different phases of methane production are better discernible regarding the first derivation after time, representing the methane production rate. The production rate was characterised by three maxima approximately at day 7, 14 and 27. Each maximum corresponds to a single phase, which could be separately described with a modified Gompertz function (Equation 1, Table 1). The first (phase I), second (phase II) and third (phase III) phase started approximately at day 0, day 10 and day 20, respectively, ending with the start of each subsequent phase or the end of the experimental period (Table 1).

**TABLE 1:** Maximum methane production rate (NmL g FF^‐1^ d^‐1^) (*R*_*m*_), maximum methane production (NmL g FF^‐1^) (*P*_*m*_), cumulative methane production (NmL g FF^‐1^) at timepoint *t* (*d*) (*P*_*t*_) and initial lag phase (*d*) (λ) as calculated via modified Gompertz equation according to Equation (1).

		Phase I			Phase II			Phase III			End of experiment			
Treatment	Aerobic incubation period [d]	R_m_ (NmLgFF^−1^d^−1^)	λ (d)	*P*_*m*_ (NmLgFF^−1^)	*R*_*m*_ (NmLgFF^−1^d^−1^)	λ (d)	*P*_*m*_ (NmLgFF^−1^)	*R*_*m*_ (NmLgFF^−1^d^−1^)	λ[d]	*P*_*m*_ (NmLgFF^−1^)	λ sum [d]	*P*_*m*_ (NmLgFF^−1^)	RMSE (NmLgFF^−1^)	*R* ^2^
Sterile	0	54.1 (1.7)	5.3 (0.1)	52.7 (6.1)	159.9 (9.2)	2.5 (0.5)	232.2 (11.8)	60.9 (2.8)	4.2 (0.2)	108.0 (18.7)	12 (0.8)	393.0 (1.8)	6.1 (0.3)	0.998
	3	49.4 (5.8)	5.3 (0.1)	47.4 (4.1)	167.7 (14.3)	2.4 (0.1)	218.0 (5.9)	64.9 (5.3)	4.1 (0.1)	112.9 (4.6)	11.8 (0.3)	378.3 (4.2)	5.6 (0.7)	0.998
	6	55.6 (6.6)	5.3 (0.4)	54.0 (2.3)	159.6 (13.6)	2.7 (0.5)	232.3 (3.5)	96.4 (91.5)	3.6 (2.1)	93.3 (5.6)	11.5 (3)	378.5 (6.6)	6.2 (2.5)	0.998
	10	54.1 (3.2)	5.4 (0)	48.9 (4.1)	157.8 (17.6)	2.7 (0.4)	221.4 (15.4)	60.9 (4.4)	3.9 (0.2)	119.9 (1.8)	12 (0.7)	390.2 (21.1)	6.4 (0.6)	0.997
	14	63.8 (5.8)	5.7 (0)	55.3 (6.4)	141.3 (13.5)	3.6 (0.9)	213.4 (17.6)	39.5 (8.8)	3.1 (1.1)	104.2 (6.2)	12.4 (2.2)	372.8 (25.5)	8.3 (1.6)	0.996
Vital	0	51.0 (4)	5.3 (0.1)	50.3 (3.9)	183.2 (19.5)	2.3 (0.2)	237.2 (16.4)	69.4 (10.4)	4.2 (0.1)	99.3 (9.8)	11.8 (0.4)	386.8 (26.5)	4.9 (0.7)	0.999
	3	44.2 (3.6)	5.1 (0.2)	51.4 (4.7)	151.3 (39.6)	1.3 (0.5)	157.1 (12.8)	118.0 (14)	3.5 (1.3)	155.8 (5.8)	9.9 (1.9)	364.2 (9)	5.0 (1.1)	0.998
	6	44.9 (4.2)	5.0 (0.1)	56.3 (5.6)	154.8 (30.2)	0.7 (0.2)	171.5 (12.1)	123.8 (59.3)	3.0 (1.2)	163.1 (11.2)	8.7 (2)	389.7 (13.6)	5.4 (0.4)	0.998
	10	46.2 (3)	5.1 (0.2)	53.9 (6.8)	161.9 (12.6)	1.1 (0.1)	172.9 (8.6)	83.5 (1.6)	2.6 (0.2)	150.9 (6.2)	8.8 (0.5)	377.7 (9.3)	5.3 (0.3)	0.998
	14	44.2 (2.2)	4.9 (0.3)	55.9 (5.9)	159.6 (4.8)	1.1 (0.3)	167.1 (26.3)	113 (16.7)	2.1 (1.84)	141.8 (6.1)	8.1 (2.4)	364.1 (16.3)	5.2 (0.7)	0.998

The three consecutive Gompertz curve fittings resulted in an overall *R* ^2^ > 0.995 and an overall root mean square error (RMSE) < 10 NmL methane g^−1^ FF. The variables describing the Gompertz functions of each methane production phase (I, II, and III) included the lag phase period (λ), the maximum methane production rate (R_m_) and the maximum cumulative methane yield (P_m_). Thereby, λ_I_, λ_II_, λ_III_, *R*_mI_, *P*_mII_ and *P*_mIII_ as well as the sum of all lag phases λ_sum_ were significantly different between filters treated with vital spores and control filters after aerobic pre‐treatment periods of ≥3 days (Table 1). These results clearly indicate that the fungal pre‐treatment affected cellulose degradation rates and decomposition kinetics during anaerobic digestion. This assumption is underlined by findings of Jeihanipour et al. (2011), who could show that chemically treated and therefore more amorphous cellulose led to higher solubilisation rates during anaerobic digestion. In the next section, the results from Gompertz modelling are discussed in more detail.

According to Gompertz fit, phase I was characterised by an extended lag phase followed by a rather low maximal methane production rate in all treatments. During this initial phase, the microbial community was probably adapting to cellulosic substrate as observed previously (Ahmed et al., 2019). In this phase, treatments with vital spores showed a reduced lag phase in comparison with control treatments, showing an accelerated initiation of methane production due to fungal pre‐treatment.

Compared with the first phase, the second phase was characterised by a higher methane production rate and a shorter lag phase. During this period the major share of the overall methane yield was produced, indicating that decomposition of the substrate was proceeding well, providing sufficient substrates for the active methanogenic community. In this phase, filters treated with vital spores showed a significantly shorter lag phase but also a significantly reduced cumulative methane yield, meaning that methanogenesis started earlier but was less productive. These findings were consistent with the results of Jeihanipour et al. who observed an increased cellulose solubilisation of more amorphous cellulose (Jeihanipour et al., 2011).

In all treatments, phase III yielded less methane than phase II, whereby the methane yield from filters treated with vital spores was significantly higher, compensating the shortage in methane production during phase II and finally resulted in an equal cumulative methane yield in all treatments after 31 days of anaerobic incubation. This last phase could be attributed to the methanogenic conversion of thermodynamically less feasible fermentation product propionic acid. This substrate probably had special metabolic requirements and an adaptation of the microbial community was needed, resulting in a prolonged lag phase.

Results from methane production kinetics suggest, that fungal pre‐treatment led to differences in substrate degradability, thus influencing degradation pathways and decomposition rates. To further investigate shifts in the metabolic pathways, intermediates of anaerobic degradation were analysed and are discussed in the next sections.

#### Intermediates of anaerobic digestion

**FIGURE 3:**
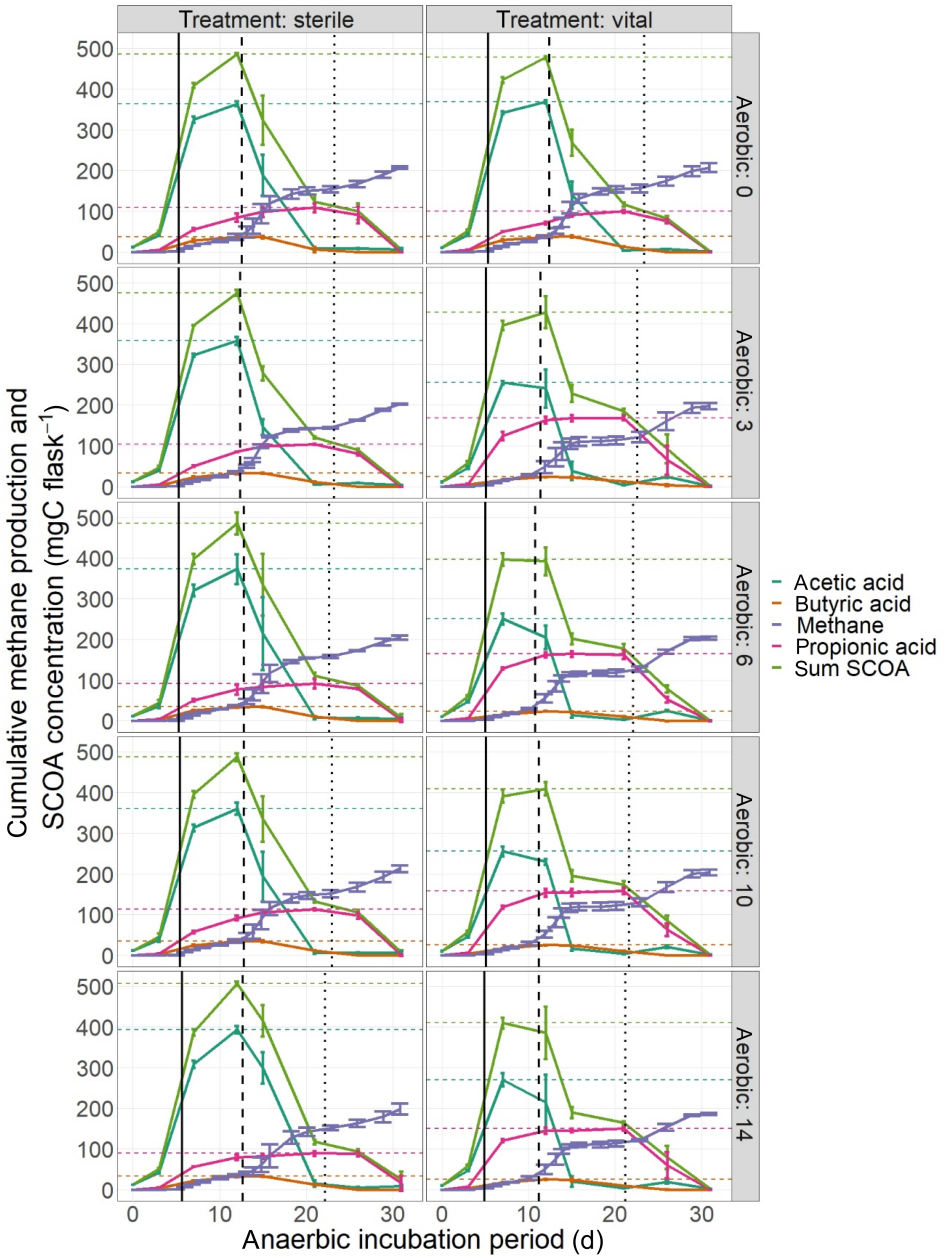
Short chain organic acid (SCOA) (mg C flask^−1^) and methane production (mg C flask^−1^) during anaerobic digestion of substate aerobically pre‐treated with sterile (sterile) and vital (vital) spores of *Trichoderma viride* for 0, 3, 6, 10 and 14 days. Horizontal lines mark the maximum for each SCOA per treatment, vertical lines indicate the start of the different biogas production phases (please also refer to Table 1).

Primary intermediates of anaerobic cellulose degradation are cellobiose (disaccharide) and subsequently glucose (monosaccharide). Via HPLC analysis, these sugars could not be detected thus reflecting a high turnover of these easily degradable compounds. During acidogenic and acetogenic processes, these sugars are mostly converted to hydrogen and carbon dioxide as well as short chained alcohols and organic acids (Leschine, 1995). Among these fermentation products, short chained organic acids (SCOA) play the central role. Their composition and concentration dynamics during anaerobic degradation are depicted in Figure 3. SCOA production was initiated immediately with the start of the anaerobic incubation but stayed at a low level until day 3. Following this initial lag phase, SCOA concentrations massively increased reaching a maximal level of 338 ± 14 mg C flask^‐^^1^ at day 7 and 398 ± 17 mg C flask^−1^ at day 12 on filters treated with vital spores and control filters, respectively. In this context, the most abundant SCOA was acetic acid followed by propionic and butyric acid (sum of *n*‐butyric and iso‐butyric acid). Shifts in SCOA dynamics caused by fungal pre‐treatment regarding these three acids were highly significant. While acetic acid in control treatments reached a maximum at day 12, the maximum level in treatments treated with vital spores was measured at day 7. Thereby, the maximum acetic acid concentration in treatments treated with vital spores reached approximately 69% of the maximum acetic acid concentration in controls. Compared with controls, filters treated with vital spores showed an increased propionic acid concentration, with the highest amount being measured at day 21 for all treatments. Thereby, propionic acid concentration reached approximately 160% in treatments treated with vital spores relative to controls. Butyric acid concentrations were comparatively low and showed significant but minor differences between treatments pretreated with sterile and vital spores.

Furthermore, overall SCOA dynamics highly corresponded to methane production. Accumulation of these fermentation products indicated high rates of cellulose hydrolysis during phase I (day 0–10) and phase II (day 10–21) (Figure 3). Further, the major share of methane production during phase II could be assigned to acetic acid consumption, while propionic and butyric acid were mostly converted into methane during phase III (day 21–31).

The comparatively lower accumulation of acetic acid in the treatments with vital spores indicated an earlier consumption, being consistent with the shortened second lag phase of methane production in these treatments. These findings could be explained again by enhanced cellulose solubilisation in treatments with vital spores. In contrast, samples treated with sterile spores showed an extended second lag phase of methane production, resulting in higher acetic acid accumulation, which finally led to higher methane yields until the end of phase II. In agreement with progression of phase II, acetic acid was exhausted at day 15 and at day 21 in treatments treated with vital and sterile spores, respectively. In contrast to acetic acid, higher concentrations of propionic acid were observed in treatments treated with vital spores and led to an increased methane yield during phase III. At the end of the anaerobic incubation, SCOA were almost completely degraded with a maximum mean concentration <10 mg C g^−1^ FF, indicating almost complete substrate conversion.

Thus, fungal pre‐treatment affected cellulose to SCOA degradability. Overall SCOA accumulation during the highly productive phase II was reduced due to an early onset of methanogenesis in the assay with vital spores. Differences in fermentation products further indicated differences in the structural composition of the cellulose due to the pre‐treatment, inducing shifts in degradation pathways and presumably the microbial community composition.

#### Electron flux from substrate into methane

**FIGURE 4:**
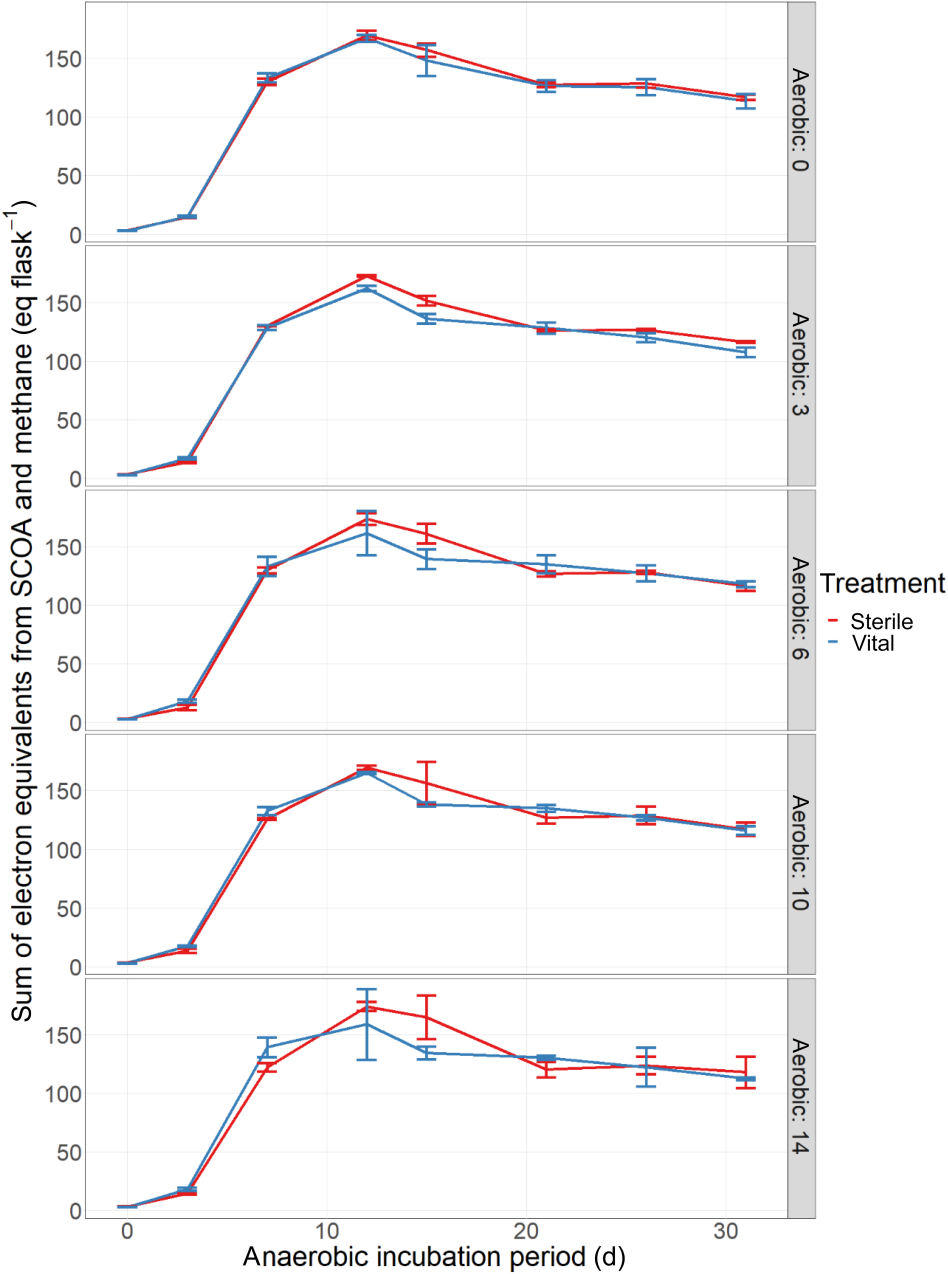
Electron flux (mean ± SD) derived from chain organic acids (SCOA) and methane during anaerobic digestion of substate not (sterile) and pre‐treated (vital) with *Trichoderma viride* for 0, 3, 6, 10 and 14 days. For calculation, the electron equivalents from a complete oxidation of the SCOAs and methane with oxygen was cumulated per timepoint.

A more theoretical strategy was pursued to investigate the overall electron flux from cellulose to SCOA and methane. It may provide a more holistic view on the effect of fungal cellulose pre‐treatment on the energy conversion during anaerobic digestion. The total amount of electron equivalents convertible into methane can be approximated by summing up the electron equivalent from cellulose and the methane amount generated by the anaerobic inoculum, resulting in 220 ± 5 meq^−^ flask^−1^. To illustrate the overall conversion of the substrate during the digestion process, the sum of electron equivalents of the main fermentation intermediates (acetic, propionic and butyric acid) and end products (methane) is depicted in Figure 4.

Substrate turn‐over was marked by the immediate SCOA production. Thereby, the highest electron equivalent amount was reached at day 12 in all treatments (phase II), marking the point of complete hydrolysis and acidogenesis during cellulose degradation. This assumption could be confirmed by visual inspection of the incubation flasks, as no filter or lint was visible anymore at this time. On day 12, 207 ± 6 meq^−^ flask^−1^ were converted when fermenting FF treated with sterile spores. The contribution of the electron equivalents of methane was 11.6 ± 0.9%. In contrast to controls, filters treated with vital spores led to a total conversion of 191 ± 15 meq^−^ flask^−1^ with a methane contribution of 18.2 ± 3.0% during the same incubation period. This means, that although less electrons were accumulated the amount of methane was higher in treatments with vital spores. The differences in maximal electron equivalents could be primarily assigned to the reduced accumulation of SCOA. This difference diminished with ongoing anaerobic digestion leading to similar levels in all treatments at the end of the experiment. Due to the higher maximum accumulation in treatments with sterile spores, the decline of converted electrons from the day with maximum to the end of the experiment was lower in treatments with vital spores. This difference might be explained by a shift of electrons from fermentation products to anabolic metabolism for biomass production and was higher in treatments with sterile spores. Thus, it can be assumed that fungal pre‐treatment promoted earlier augmentation of anaerobic biomass and catalytic activity, resulting in a reduced SCOA accumulation and an accelerated methanogenic activity during phase I and II of the anaerobic digestion.

### Dynamics of community composition during anaerobic digestion

Prior analysis of the pre‐treatment effect, the temporal dynamics of the microbial community composition during the batch fermentation of cellulose filters was evaluated. For this purpose, the treatment with sterile spores at day 0 was chosen. The anaerobic degradation process depends on the cooperation of a microbial consortium with different members being engaged in different degradation steps. During batch digestion, these steps take place sequentially and active taxa change their relative abundance during time. We are aware that the compositional character of data and varying 16S rRNA gene copy numbers, do not allow statements on the absolute abundance of single taxa. Nevertheless, the proportion of all reads occupied by a single taxon still gives valuable information on its role within the system and the community. Therefore, 154 significantly differential OTUs were determined over incubation time. The following discussion concentrates on those taxa with relative abundances reaching at least 1% at one sampling point (*n* = 21).

OTUs, which show their maximum relative abundance at the beginning of the incubation mainly represented the microbial community of the inoculum. These were members of the genera Candidatus_*Cloacimonas*, *Fastidiosipila*, *Bacteroidetes*_vadinHA17_NA, and Subgroup_10 (a Genus within the *Thermoanaerobaculaceae*). Among the others, eleven OTUs could be classified as fermenting organisms producing volatile fatty acids (VFAs), H_2_ and CO_2_. They comprised the genera *Acholeplasma*, WWE3_NA (a class within the Patescibacteria), *Anaerolineaceae*_NA, *Anaerolineaceae*_NA, Candidatus_*Caldatribacterium*, *Longilinea*, *Flexilinea*, *Hydrogenispora*_NA and YC‐ZSS‐LKJ63 (a genus within the *Hydrogenedensaceae*), all showing maximal relative abundance between day 21 and 31. Especially noteworthy was *Longilinea* sp. with up to 15.0% of relative abundance—the highest value for a single OTU in this data subset. The genus *Longilinea* contains only one described species *Longilinea arvoryzae* which was previously found to produce acetate, lactate and hydrogen and to be supported by *Methanospirillum hungatei* in co‐culture (Yamada et al., 2007).

Regarding syntrophic organisms, OTU 142_Pelotomaculum, a genus known for syntrophic propionate oxidation, was present only towards the end of the incubation with a maximum relative abundance of 1.5% at day 31. Further, OTU 1153_Syntrophomonas was also among the significantly differential OTU between the treatments and showed an increase in relative abundance in the second half of incubation with a maximum at day 26 (1.0%). The genus *Syntrophomonas* is capable of oxidising several fatty acids in syntrophic co‐operations with methanogens (Wu et al., 2005). As the propionate pool was depleted during the same time, an involvement of both OTUs (142 and 1153) in propionate oxidation is assumed.

Of all members of the community, methanogenic *Archaea* were the only ones which could undoubtedly be assigned to methanogenesis. The dominant methanogenic OTUs at the beginning of the incubation were 500_Methanosaeta and 501_Methanosaeta, with 11.6% and 1.5% reached at day 3 and 0, respectively. *Methanosaeta* sp. are strictly acetoclastic methanogens specialised to use low acetate concentrations (Ferry, 2010; Liu & Whitman, 2008; Smith & Ingram‐Smith, 2007; Welte & Deppenmeier, 2014; Westerholm et al., 2016). This is plausible as the inoculum derived from a large‐scale biogas plant of a wastewater treatment facility with low acetate concentrations during stable operation (Aichinger et al., 2015). As acetate concentrations were generally higher in batch reactors of this study (Table 1), the relative abundance of *Methanosaeta* sp. quickly declined and that of *Methanosarcina* sp. increased over time, with a maximum relative abundance of 14.9% on day 15 and 6.9% on day 31. *Methanosarcina* spp. is a more efficient acetate consumer with a wider substrate range than *Methanosaeta* spp. (Ferry, 2010; Liu & Whitman, 2008; Lovley, 2018; Welte & Deppenmeier, 2014).

Towards the end of the incubation, the only remaining VFA was propionate. Propionate oxidation is the energetically least favourable VFA oxidation reaction during anaerobic degradation and engages organisms strictly depend on syntrophic partners (Hao et al., 2020; Singh et al., 2021; Westerholm et al., 2021). Most of the time, propionate is only depleted when acetate and butyrate concentrations are low. In most cases, propionate oxidation results in the production of acetate and H_2_ (Worm et al., 2014; Jan Dolfing, 2013), both consumable by *Methanosarcina* sp. (Lackner et al., 2018); however, H_2_ could also be consumed by other chemolithotrophs like hydrogenotrophic methanogens, for example, *Methanobacterium* spp., which were present but less abundant during the entire process presented here.

### Effect of pre‐treatment on anaerobic community

**FIGURE 5:**
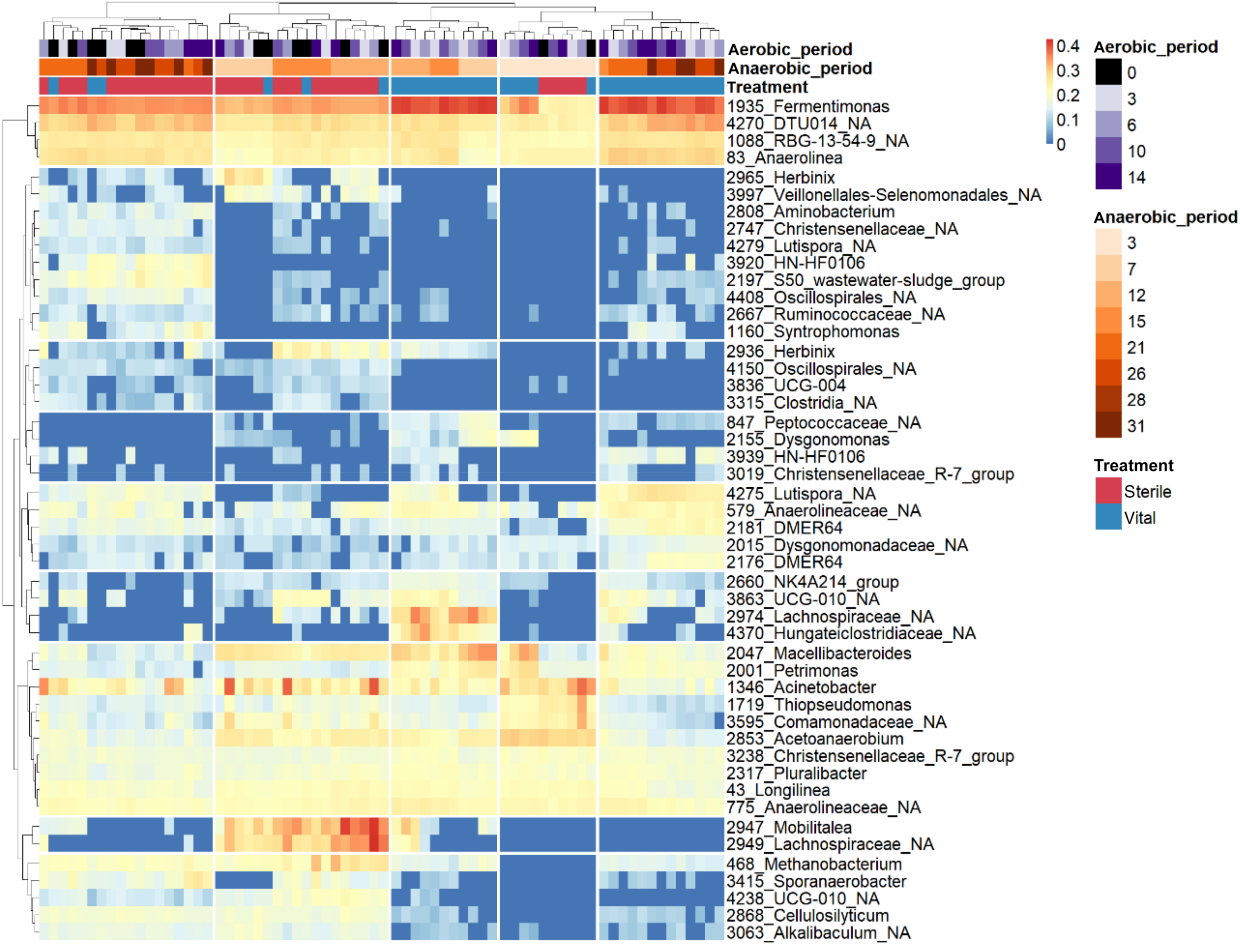
Abundance of 53 operative taxonomic units (OTUs) significant for the difference between treatment with sterile and vital spores. The abundance gradient is depicted by the colour gradient blue‐yellow‐red. Euclidean distance measure was used for clustering rows and columns.

The effect of the fungal pre‐treatment on the anaerobic microbial community composition during the entire digestion period was tested using individual datasets for each pre‐treatment period. For each of these datasets, comparisons for the treatment with sterile versus vital spores were conducted. The ANCOM‐BC analysis of these five datasets resulted in 21, 28, 21 and 20 significantly differential taxa for a pre‐treatment period of 3, 6, 10 and 14 days, respectively. Nine of these OTUs were repeatedly found by all four separate analyses, meaning that they represent a very stable answer to the pre‐treatment independently of the duration of the pre‐treatment. In total, 53 different OTUs marked the difference between treatment with sterile and vital spores. The abundance of these OTUs is depicted in a heatmap in Figure 5. Among them, OTUs positively affected by the pre‐treatment were members of the genera *Lutispora*, *Fermentimonas*, *Petrimonas* and *Dysgonomonas*. In contrast, negatively affected OTUs belonged to the genera *Cellulosilyticum*, *Sporanaerobacter*, S50_wastewater‐sludge_group, *Alkalibaculum*, and UCG‐010_NA. *Lutispora thermophila*, the only species of its genus so far, is described to be proteolytic and unable to use carbohydrates and was isolated from a thermophilic biogas reactor (Shiratori et al., 2008). Therefore, it can be assumed that the presence of *Lutispora* spp. was supported by proteinaceous parts of fungal biomass and its intermediates. In contrast, *Fermentimonas* sp. and *Petrimonas* sp. are known to ferment proteins and carbohydrates. They show weak enzymatic activity against cellobiose and CM‐cellulose (carboxymethyl‐cellulose), but are unable to degrade crystalline cellulose (Hahnke et al., 2016). Besides cellobiose, *Fermentimonas* sp. are also able to degrade di‐ and monosaccharides (Beye et al., 2018), thus pre‐treatment increased substrate availability for this genus. Members of the genus *Dysgonomonas* found to be positively affected by the fungal pre‐treatment; they are sugar fermenting organisms often showing α‐glucosidase and β‐glucosidase activity (Hofstad et al., 2000; Kita et al., 2015). On the contrary, *Cellulosilyticum* sp., named after its ability to anaerobically degrade cellulose, was merely found in pretreated samples and if in very low abundances indicating that the fungus changed the amount and/or structure of the available cellulose (Cai & Dong, 2010) leaving no suitable substrates for members of this genus. The only cultivated member of the genus *Sporanaerobacter* was described to ferment glucose to acetate and peptone to mainly acetate, H_2_ and CO_2_ (Hernandez‐Eugenio et al., 2002). The reasons for the inhibition of this genus in the pre‐treated samples are speculative and may be caused by competitive effects. In samples with sterile spores, *Alkalibaculum* sp. occurred with two peaks in its relative abundance at day 7 and 26 of the anaerobic digestion. The known species of this genus are both acetogenic (Allen et al., 2010; Khomyakova et al., 2020). The metagenome analysis derived OTUs S50_wastewater‐sludge_group and UCG‐010_NA were also inhibited by the pre‐treatment with vital fungi and showed their maximum relative abundances at the end of day 12/15 of digestion. Further information on these taxa is not available. All in all, the ANCOM‐BC analysis showed that single taxa were strongly affected in their ability to occupy their niche by the pre‐treatment with an active *T. viride*.

## CONCLUSION

Biological pre‐treatment of cellulose with the soft‐rot‐fungus *T. viride* had a significant impact on downstream anaerobic digestion. Fungal growth and cellulolytic activity caused by the aerobic pre‐treatment were clearly proven, and a significant effect on methane production kinetics was found; however, total methane yield was not significantly affected by the applied pre‐treatment. Further, *T. viride* pre‐treatment might have induced an earlier augmentation of anaerobic biomass and an accelerated methanogenic activity, resulting in a reduced accumulation of fermentation intermediates during batch cultivation. Therefore, fungal pre‐treatment affected substrate degradability, thus influencing the degrading microbial community and decomposition rates during anaerobic digestion. The differences in intermediate accumulation were reflected by dynamics in the microbial community composition, with maximal relative abundances of syntrophic and methanogenic taxa occurring in the later phases of the incubation period. The pre‐treatment of the cellulose filters for 3 to 14 days had a significant effect on a range of taxa comprising mostly fermenting bacteria.

The present study contributes to a better understanding of fungal pretreatment of cellulose (residues) mainly impacting the kinetics of methane production and the anaerobic microbial community; however, applying substrates including not only cellulose but also lignin compounds (lignocellulose, “real substrates”) still has potential for further optimisation and investigations.

## AUTHOR CONTRIBUTIONS

**Rudolf Markt:** Conceptualization (equal); formal analysis (equal); funding acquisition (equal); investigation (equal); methodology (equal); writing – original draft (equal). **Eva Maria Prem:** Data curation (supporting); validation (supporting); visualization (supporting); writing – review and editing (lead). **Nina Lackner:** Validation (supporting); writing – review and editing (supporting). **Mira Mutschlechner:** Conceptualization (supporting); writing – review and editing (equal). **Paul Illmer:** Conceptualization (supporting); resources (equal). **Andreas Otto Wagner:** Conceptualization (equal); data curation (supporting); funding acquisition (lead); methodology (supporting); project administration (lead); resources (lead); supervision (lead); validation (equal); writing – review and editing (equal).

## CONFLICT OF INTEREST STATEMENT

The authors declare no conflicts of interest.

## Data Availability

The raw sequence reads can be found on the NCBI under BioProject PRJNA997780: https://www.ncbi.nlm.nih.gov/bioproject/997780.
